# Evolution of pigment synthesis pathways by gene and genome duplication in fish

**DOI:** 10.1186/1471-2148-7-74

**Published:** 2007-05-11

**Authors:** Ingo Braasch, Manfred Schartl, Jean-Nicolas Volff

**Affiliations:** 1 University of Würzburg, Physiological Chemistry I, Biozentrum, Am Hubland, 97074 Würzburg, Germany; 2 Institut de Génomique Fonctionnelle, Université de Lyon, F-69003, France, INRA; CNRS, Université Lyon 1, Ecole Normale Supérieure, F-69364, France

## Abstract

**Background:**

Coloration and color patterning belong to the most diverse phenotypic traits in animals. Particularly, teleost fishes possess more pigment cell types than any other group of vertebrates. As the result of an ancient fish-specific genome duplication (FSGD), teleost genomes might contain more copies of genes involved in pigment cell development than tetrapods. No systematic genomic inventory allowing to test this hypothesis has been drawn up so far for pigmentation genes in fish, and almost nothing is known about the evolution of these genes in different fish lineages.

**Results:**

Using a comparative genomic approach including phylogenetic reconstructions and synteny analyses, we have studied two major pigment synthesis pathways in teleost fish, the melanin and the pteridine pathways, with respect to different types of gene duplication. Genes encoding three of the four enzymes involved in the synthesis of melanin from tyrosine have been retained as duplicates after the FSGD. In the pteridine pathway, two cases of duplicated genes originating from the FSGD as well as several lineage-specific gene duplications were observed. In both pathways, genes encoding the rate-limiting enzymes, tyrosinase and GTP-cyclohydrolase I (GchI), have additional paralogs in teleosts compared to tetrapods, which have been generated by different modes of duplication. We have also observed a previously unrecognized diversity of *gchI *genes in vertebrates. In addition, we have found evidence for divergent resolution of duplicated pigmentation genes, *i.e*., differential gene loss in divergent teleost lineages, particularly in the tyrosinase gene family.

**Conclusion:**

Mainly due to the FSGD, teleost fishes apparently have a greater repertoire of pigment synthesis genes than any other vertebrate group. Our results support an important role of the FSGD and other types of duplication in the evolution of pigmentation in fish.

## Background

Coloration and color patterning of skin, scales, feathers, and hair belong to the most diverse phenotypic traits in vertebrates and have a plethora of functions such as camouflage, warning or threatening of predators, and species recognition [1,2]. Coloration is the result of diverse pigments synthesized by pigment cells or chromatophores, which are derived from the neural crest. There are noticeable differences in the number of chromatophore types among vertebrate groups. Mammals and birds possess only the brown to black melanocytes, while amphibians and reptiles additionally have the yellow to red xantho-/erythrophores and the reflecting iridophores. In teleost fish, up to five different pigment cell types have been identified, with white leucophores and blue cyanophores in addition to the aforementioned cell types (reviewed in [2]). Some pigment cell types in teleosts are even further partitioned into distinct sublineages that are under different genetic control [3,4].

The genetic basis of pigment cell development and differentiation is largely conserved between mammals and teleosts. Many genes such as *Sox10*, *Mitf*, *Kit *and *Ednrb*, some of them first identified through the cloning of coat color mutations in mice, have subsequently been found to be involved in pigmentation in teleost fish as well [5-8]. Other genes with functions in pigmentation like *slc24a5 *were identified first in teleosts and later on in mammals [9]. However, an important difference between teleost fish and tetrapods has recently emerged from several studies on particular fish species. For some single copy pigmentation genes of tetrapods, two paralogous genes are present in teleost genomes, possibly as the result of a fish-specific whole-genome duplication (FSGD) that occurred ~250 to 350 million years ago (mya) in a common ancestor of teleosts (reviewed in [10-13]). Examples of such duplicated genes include *sox10a *and *sox10b *[14], *mitfa *and *mitfb *[15,16], *kita *and *kitb *[17], *csf1ra *and *csf1rb *[18] and *pomca *and *pomcb *[19], for which at least one of the duplicates has been shown to participate in pigment cell development in fish. These genes encode transcription factors (*sox10*, *mitf*), signaling molecules (*pomc*) or cell-surface receptors (*kit, csf1r*) and are involved in neural crest specification (*sox10*) or commitment of pigment cell precursors to a particular chromatophore fate (*mitf, kit*: melanophores; *csf1r*: xanthophores).

A major step in chromatophore differentiation is the biosynthesis of the pigment displayed by the respective type of pigment cells. Although there are sporadic reports of duplicated genes for pigment synthesis enzymes in specific teleost lineages [20-22], no systematic genomic analysis has been performed so far to determine the complete set of duplicated pigmentation genes in fish and to better understand how pigment synthesis pathways as a whole have been affected by the FSGD.

In the present studies, we have analyzed genes involved in the biosynthesis of the dark pigment melanin, which is produced by melanophores [23,24], and of the pteridine pigments synthesized in xanthophores (reviewed in [25]). We find that the FSGD had a deep impact on the melanin synthesis pathway, with three out of four enzyme-encoding genes being duplicates in teleosts. The pteridine synthesis pathway has been affected to a lesser degree by the FSGD, with two of nine enzymes represented by two teleost-specific paralogs. Several cases of lineage-specific duplication were also observed in the pteridine pathway. In both pathways, genes encoding the rate-limiting enzymes are duplicated in teleosts compared to tetrapods, with different modes of duplication being involved.

## Results

Phylogenetic analyses and synteny studies were applied to the reconstruction of the evolution of genes involved in pigmentation pathways in ray-finned fish. The combination of these complementary approaches is particularly helpful to deduce the evolutionary history of gene families following gene and genome duplication events (reviewed in [26]). Most genes duplicated during large-scale and in particular whole genome duplications are part of so-called paralogons, *i.e*., chromosomal blocks of two or more duplicated (paralogous) genes ([18] and references therein). The detection of such paralogons helps to draw conclusions on the origin of duplicated genes when phylogenetic signals are not informative. In the present study, sequence information from the genome assemblies of five teleost species – two pufferfish species (*Tetraodon nigroviridis, Takifugu rubripes*), medaka (*Oryzias latipes*), three-spined stickleback (*Gasterosteus aculeatus*) and zebrafish (*Danio rerio*) – and from diverse teleost expressed sequence tag (EST) resources have been compared to data from tetrapods (human, mouse, chicken, frog) and different invertebrate outgroups (urochordates, sea urchin, fruitfly, nematodes). The results of these surveys are summarized in Table 1; accession numbers are given in the Additional files [see Additional files 1, 2].

**Table 1:** Pigment synthesis genes in human and teleost fish

**gene**	**human**	**zebrafish**	**medaka**	**stickleback**	**Takifugu**	**Tetraodon**	**other teleosts**
***tyr^#^***	chr 11	LG 15 (*sdy*)	LG 13 (*i-b/-1/-4/-6*)	chr I	scaf 172	scaf 12050	Omy, Oni and other cichlids
		-	chr 14	chr VII	scaf 176	chr 7	Eha, Gpe, Ipu, Omy, Oni
***tyrp1^#^***	chr 9	LG 7*	LG 18	chr VII	scaf 2235	scaf 14681	Cau, Omy, Oni, Ppr, Ssa
		LG 1	chr 1	chr IX	-	scaf 13631^ψ^	Man, Ppr
***dct***	chr 13	LG 9	chr 21	chr XVI	scaf 103	chr 2	Cau, Ipu, Omy, Ssa
***silv^#^***	chr 12	LG 11 (*fdv*)	chr 5	scaf 27	scaf 47	chr 11	Hsp, Omy, Ppr
		chr 23	chr 7	chr XII	scaf 304	chr 9	Abu, Omy, Ssa
***oca2***	chr 15	chr 6	LG 4 (*i-3*)	chr VIII	scaf 13	chr 1	Ame
***aim1***	chr 5	LG 21	LG 12 (*b*)	chr XIV	scaf 44	chr 4	Omy, several cichlids
***slc24a5***	chr 15	LG 18 (*gol*)	chr 3	chr II	scaf 1	chr 5	Ppr

***gchIa***	chr 14	LG 17*	scaf445	chr II	scaf 1	scaf 15099	Hhi, Omy, Ppr
***gchIb***	-	LG 12*	chr 19	chr V	scaf 952	scaf 7971	Fhe
***gchIc***	-	-	-	chr VIII	scaf 208	chr 1	Sau
***gchfr***	chr 15	chr 17	chr 22	chr XV	scaf 23	chr 10	Fhe, Omy, Ssa
***pts***	chr 11	chr 12	chr 19	chr V	scaf 3	scaf 14338	Omy, Pfl, Ppr, Pre, Ssa
***spr^#^***	chr 2	chr 5	chr 10	chr XIV	scaf 106	scaf 21575	Ipu, Omy, Pfl, Pol, Ppr, Ssa
		chr 8	-	chr XIII	scaf 11	-	-
***xdh***	chr 2	LG 22	chr 2	chr I	scaf 57	chr 3	Fhe, Sch, Ssa
***clot (txnl5)***	chr 17	chr 15	chr 13	chr I	scaf 271	chr 16	Cca, Ipu, Omy, Ppr, Ssa
***pcbd1***	chr 10	chr 13	chr 14	chr VI	scaf 53, scaf 178^ψ^	chr 17	Fhe, Hhi, Ipu, Omy, Pfl
***pcbd2***	chr 5	chr 21	chr 15	scaf 324	scaf 71	chr 7	Man, Omy, Ppr, Ssa
***dhpr^#^***	chr 4	chr 14	chr 10	chr IV	scaf 70	chr 20	Cca, Omy, Ppr, Psa, Ssa, Hsp
		chr1	-	-	-	-	Omy, Ppr, Ssa
***pam***	chr 13	LG 9 (*esr*)	chr 21	chr XVI	scaf 31	chr 2	Abu, Hhi, Omy, Ppr

Genomic locations are given as linkage groups (LG) if genes have been mapped or as chromosomes (chr) or scaffolds (scaf) as obtained from Ensembl genome assemblies [77]. Three zebrafish genes have been mapped with radiation hybrid panel in the present study (*). Names of mutants from zebrafish and medaka are given in brackets. ^ψ ^indicates pseudogenes. # indicates duplications based on the fish-specific genome duplication, lineage-specific duplications are underlined. Scientific names of other teleosts are abbreviated as follows: Abu: *Astatotilapia burtoni *(haplochromine cichlid); Ame: *Astyanax mexicanus *(Mexican tetra); Cau: *Carassius auratus *(goldfish); Cca: *Cyprinus carpio *(common carp); Eha: *Elops hawaiensis *(Hawaiian ladyfish); Fhe: *Fundulus heteroclitus *(killifish); Gpe: *Gnathonemus petersii *(elephantnose fish); Hsp: *Haplochromis *spec. (haplochromine cichlid); Hhi: *Hippoglossus hippoglossus *(Atlantic halibut); Ipu: *Ictalurus punctatus *(channel catfish); Man: *Misgurnus anguillicaudatus *(oriental weatherfish); Omy: *Oncorhynchus mykiss *(rainbow trout); Oni: *Oreochromis niloticus *(Nile tilapia); Pfl: *Platichthys flesus *(European flounder); Pol: *Paralichthys olivaceus *(bastard halibut); Ppr: *Pimephales promelas *(fathead minnow); Pre: *Poecilia reticulata *(guppy); Psa: *Pomatomus saltatrix *(bluefish); Sau: *Sparus aurata *(gilthead seabream); Sch: *Siniperca chuatsi *(Chinese perch); Ssa: *Salmo salar *(Atlantic salmon).

### Gene duplications in the melanin synthesis pathway

**Figure 1:**
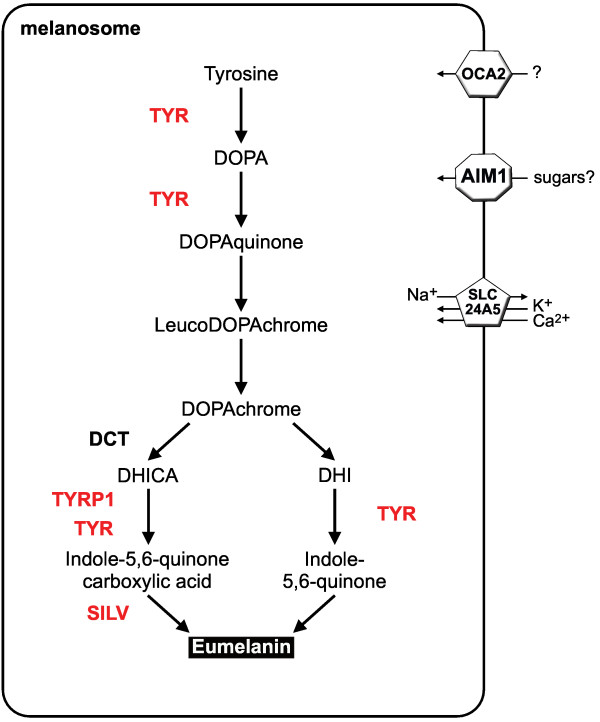
**Eumelanin synthesis pathway and gene duplications in vertebrates**. Eumelanin is synthesized from tyrosine within the melanosome of melanophores. This requires members of the Tyrosinase family (TYR, DCT, TYRP1) and probably Silver (SILV). Three melanosomal transporters (OCA2, AIM1 and SLC24A5) are crucial for proper melanin synthesis. Red indicates duplications during the fish-specific genome duplication.

Melanin, the dark pigment of melanophores, is synthesized from tyrosine within a specialized organelle, the melanosome [23,24]. In mammals and birds, two types of melanin are produced, the black to brown eumelanin and the lighter pheomelanin, but in teleost fishes only eumelanin has been observed [1]. The eumelanin synthesis pathway is presented in Figure 1. Disruption of melanogenesis leads to reduced pigmentation intensity culminating in complete albinism [27].

#### Tyrosinase gene family

Vertebrate melanin synthesis involves the members of the tyrosinase gene family: *tyrosinase *(*tyr*), *tyrosinase-related protein 1 *(*tyrp1*) and *dopachrome tautomerase *(*dct*; also known as *tyrosinase-related protein 2*) [23,24]. Tyrosinase (EC 1.14.18.1) promotes the first two rate-limiting steps of melanin synthesis from tyrosine to DOPA and DOPAquinone as well as two later steps. Dct (EC 5.3.3.12) converts DOPAchrome to DHICA, and Tyrp1 is involved in the formation of indole-5,6-quinone carboxylic acid from DHICA (Figure 1). During the early evolution of the chordate lineage, an ancestral *tyrosinase *gene was duplicated before the divergence of urochordates and vertebrates leading to *tyrosinase *and a *tyrosinase-related *gene. The latter one was subsequently duplicated in the vertebrate lineage giving rise to *tyrp1 *and *dct *[28,29].

**Figure 2:**
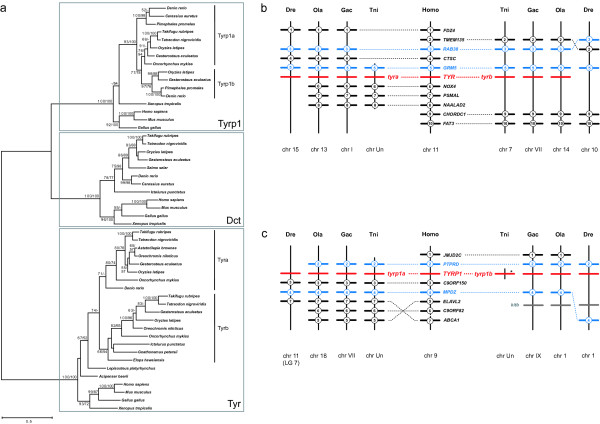
**Evolution of the tyrosinase gene family in vertebrates**. (a) Maximum-likelihood phylogeny of protein sequences from the tyrosinase family based on 570 AA positions. The tree is mid-point rooted. Numbers at the branches denote bootstrap values (maximum likelihood/neighbor joining). Bootstrap values above 50 are shown. Tyrp1a and Tyrp1b are assigned according to the analysis of their genomic environment. (b) Synteny of *tyr*-containing regions in vertebrate genomes. The human *TYR *region is syntenic to two *tyr *paralogons in Tetraodon (Tni), stickleback (Gac) and medaka (Ola). *Tyrb *was apparently lost in the zebrafish (Dre). (c) Synteny of *tyrp1*-containing regions in vertebrate genomes. The human *TYRP1 *region is syntenic to two *tyrp1 *paralogons in stickleback, medaka and zebrafish. A *tyrp1b *pseudogene is found in Tetraodon (asterisk). Numbered bars represent genes contributing to conserved synteny, genes that do not contribute to conserved synteny are not shown. Blue bars indicate genes that are duplicated along with *tyr *or *tyrp1b*, respectively. Dotted lines connect orthologous genes. *Kitb *(grey bars), another teleost-specific pigmentation gene duplicate [17] is found 3' of *tyrp1b *in the four teleost genomes but belongs to a different paralogon (see text).

The phylogeny of the tyrosinase gene family in vertebrates is presented in Figure 2a. For *tyr *and *tyrp1*, duplications were observed in teleosts, while *dct *is present as a single copy in teleosts just like in tetrapods. As shown previously, *tyr *was duplicated in teleosts during the course of the FSGD after divergence from the more basal actinopterygian lineages of sturgeon (*Acipenser baerii*) and gar (*Lepisosteus platyrhynchus*) leading to two *tyr *copies in Takifugu and cichlids [20]. Consistently, two *tyr *paralogs were found in pufferfishes, stickleback, medaka and rainbow trout. In the zebrafish, however, only the *tyra *paralog was detected, suggesting that *tyrb *has been lost. The two *tyr *paralogs previously reported for the tetraploid rainbow trout (*Oncorhynchus mykiss*) [22] are – according to the present phylogeny – the result of the FSGD rather than of another round of genome duplication in the ancestor of salmonid fishes [30,31]. Furthermore, synteny data also provide strong evidence for the duplication of *tyr *during the course of the FSGD. In human, *TYR *is found on chromosome 11q14-q21 and the (co-)orthologs of nearby genes are also found in close proximity to the *tyr *paralogs in pufferfishes, stickleback, medaka and zebrafish (Figure 2b). Most of these genes are present on one of the two paralogous chromosomal blocks (paralogons) in teleost genomes, but two genes, *grm5 *and *rab38*, are also present in duplicate. The presence of these paralogons in diverse teleost genomes is consistent with the duplication of *tyr *during the course of the FSGD. This is also in agreement with previous studies showing that the *tyr *gene-containing chromosomes 13 and 14 from medaka as well as chromosomes 15 and 10 from zebrafish (Figure 2b) are derived from the same protochromosome [32-34].

Furthermore, our analysis demonstrated that *tyr *is not the only gene found from the melanin pathway to be duplicated in fish. Two paralogs of *tyrp1 *were identified in medaka, zebrafish, stickleback and fathead minnow (*Pimephales promelas*), while only one complete *tyrp1 *paralog (*tyrp1a*) was detected in pufferfishes. In Tetraodon, additionally a region in scaffold 13631 with partial but significant sequence similarity to *tyrp1b *was found. However, some splice sites of this sequence are degenerated and the putative coding sequence contains a stop codon. We confirmed the presence of this stop codon by sequencing of genomic DNA [GenBank: EF183530], thus excluding the possibility of a sequencing error. Hence, this sequence represents most likely a *tyrp1b *pseudogene.

In the phylogeny of the entire tyrosinase gene family based on protein sequences (Figure 2a), the tree topology is not consistent with a duplication of *tyrp1 *during the FSGD. In contrast, a separate maximum likelihood phylogeny of vertebrate *tyrp1 *genes based on nucleotide sequences suggests the duplication of *tyrp1b *during the course of the FSGD [see Additional file 3]. This was also confirmed by synteny data (Figure 2c), which are generally considered as more reliable than molecular phylogenies to reconstruct large-scale duplication history [26]: the region of human chromosome 9p23 containing *TYRP1 *is syntenic to two *tyrp1*-containing paralogons in medaka, stickleback and zebrafish. Accordingly, the respective medaka chromosomes 1 and 18 have been shown to contain large duplicated segments having been formed from a same protochromosome by the FSGD [32].

In zebrafish, the previously described paralog *tyrp1b *is found on chromosome 1 in the present genome assembly (Zv6) and was mapped to the corresponding linkage group (LG) 1 [33]. The newly found *tyrp1a *paralog is found in Zv6 on chromosome 11, but was not genetically mapped so far. As a paralogous relationship between zebrafish chromosomes 1 and 11 has not been reported so far and since there are frequent discrepancies between mapping data of zebrafish genes and their chromosomal assignment in current genome assemblies ([35] and own observations), we mapped *tyrp1a *using the radiation hybrid panel LN54 [36]. The *tyrp1a *gene was assigned not to LG 11 (as expected from Zv6 genome assembly analysis) but to LG 7 at a distance of 0.00 cR from marker Z21714 with a LOD score of 27.4. However, a paralogous LG1–LG7 relationship has also not been reported for zebrafish so far. These data suggest the presence of a newly identified paralogon in the zebrafish genome. *Kitb*, another pigmentation gene duplicate that has its origin in the FSGD [17], is found 3' of *tyrp1b *(Figure 2c). However, this gene is not part of the *tyrp1 *paralogon, as *kita *is found on LG 20 and not on LG 7 in zebrafish and the human *KIT *is found on chromosome 4 and not on chromosome 9.

#### Silver

**Figure 3:**
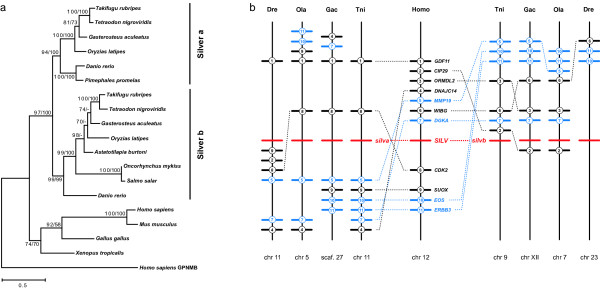
**Evolution of the *silver *genes in vertebrates**. (a) Maximum-likelihood phylogeny of Silver protein sequences based on 523 AA positions. The repeat region [21] was excluded from the alignment. The tree was rooted with human GPNMB. Numbers at the branches denote bootstrap values (maximum likelihood/neighbor joining) above 50%. (b) Synteny of *silv*-containing regions in vertebrate genomes. The human *SILV *region is syntenic to two *silv *paralogons in Tetraodon (Tni), stickleback (Gac), medaka (Ola) and zebrafish (Dre). Numbered bars represent genes contributing to conserved synteny, genes that do not contribute to conserved synteny are not shown. Blue bars indicate genes that are duplicated along with *silv*. Dotted lines connect orthologous genes.

A later step in melanin synthesis from indole-5,6-quinone carboxylic acid to eumelanin is presumably catalyzed by the Silver protein (also known as Pmel17) [37,38] (Figure 1). A recent study identified two paralogs of *silver *(*silv*) in the zebrafish [21]. We reconstructed the evolution of the *silv *gene in the vertebrate lineage and additionally found two paralogs of *silv *in both pufferfishes as well as in medaka and stickleback, with a tree topology consistent with a duplication in the course of the FSGD (Figure 3a). This conclusion is once again strongly supported by synteny data (Figure 3b): human *SILV *is found on chromosome 12q13-q14, a region that is highly syntenic to *Tetraodon *chromosomes 9 and 11, medaka chromosomes 5 and 7 and zebrafish chromosomes 11 and 23, which contain the *silv *paralogons. As all these chromosomes evolved from a same protochromosome through duplication during the FSGD [32-34,39], it is most likely that the *silv *duplicates in teleosts stem from this event.

#### Melanosomal transporters

Three non-related genes, *oca2*, *aim1 *and *slc24a5*, encode for transporter proteins residing in the melanosomal membrane and being essential for melanin synthesis (Figure 1). Loss-of-functions mutations in these genes lead to reduced melanin pigmentation in teleosts and mammals [9,40-42]. In contrast to *tyr*, *tyrp1 *and *silv*, all three transporters are encoded by a single gene in the teleost species analyzed [see Additional file 4].

### Gene duplications in the pteridine synthesis pathway

**Figure 4:**
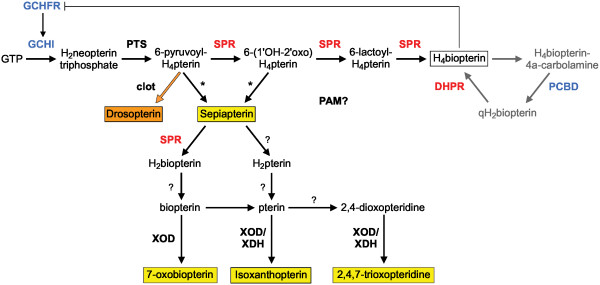
**Pteridine synthesis pathway and gene duplications in vertebrates**. Pteridine synthesis contains three component pathways [25]: the *de novo *synthesis of H_4_biopterin from GTP (top line), the H_4_biopterin regeneration pathway (grey) and the synthesis of yellow pteridine pigments. The formation of orange drosopterin has not been elucidated yet in vertebrates. In *Drosophila*, the clot enzyme is involved [53], which corresponds to the vertebrate Txnl5 protein. Asterisks indicate hypothetical reactions and question marks unidentified enzymes. Red indicates duplications during the fish-specific genome duplication, blue other types of duplication.

The yellow to reddish pteridine pigments of xanthophores are synthesized from GTP through the pteridine synthesis pathway (Figure 4) (reviewed in [25]). The pteridine pathway is composed of three component pathways. The first one leads to the *de novo *synthesis of tetrahydrobiopterin (H_4_biopterin) from GTP. H_4_biopterin is a cofactor for neurotransmitter synthesis and tyrosinase activity in melanophores [25]. The second component is the regeneration pathway of oxidized H_4_biopterin after it has acted as cofactor [25]. The third pathway shares several steps with the first one and leads to the formation of the yellow pigments, sepiapterin and its derivatives, as well as probably to the reddish drosopterin, which is also found in teleost fishes, especially in poecilids [43].

#### GTP cyclohydrolase I and its feedback regulatory protein

**Figure 5:**
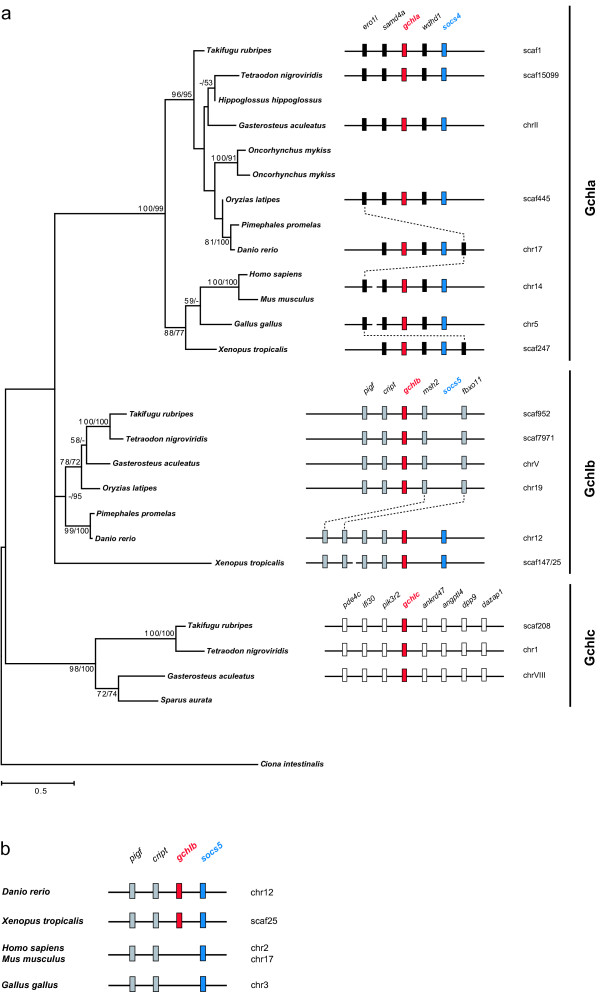
**Evolution of the GTP-cyclohydrolase I gene family in vertebrates**. (a) Maximum-likelihood phylogeny of GchI protein sequences based on 268 AA positions (left). The tree is rooted with GchI from urochordates. Numbers at the branches denote bootstrap values (maximum likelihood/neighbor joining) above 50%. Groups (GchIa, GchIb, GchIc) were assigned according to genomic environment of *gchI *genes (right). *GchIa *and *gchIb *are both linked to members of the *socs *gene family (blue). *GchIb *is absent from mammalian and avian genomes, *gchIc *is only found in some teleost lineages. Dotted lines connect orthologous genes. (b) The *gchIb *region of teleosts and amphibian is syntenic to a chromosomal block in the genomes of mammals and bird lacking *gchIb*, suggesting that *gchIb *was lost secondarily in these lineages.

The first, rate limiting step in pteridine synthesis is catalyzed by the GTP cyclohydrolase I (GchI; EC 3.5.4.16) (Figure 4). *GchI *expression is an initial step for melanophore and xanthophore differentiation due to its involvement in the different component pathways [25]. Only one *gchI *gene has been reported so far in vertebrates, including the teleosts rainbow trout [44] and zebrafish [45]. The present survey for *gchI *sequences in vertebrates, however, revealed an unexpected diversity of *gchI *genes in teleosts and amphibians (Figure 5). While a single *gchI *gene was found in mammals and birds, two *gchI *genes were identified in frog, medaka, zebrafish, and fathead minnow, and even three genes are present in pufferfishes and stickleback. Taking conserved syntenies into account, three groups of *gchI *genes became obvious.

**Figure 6:**
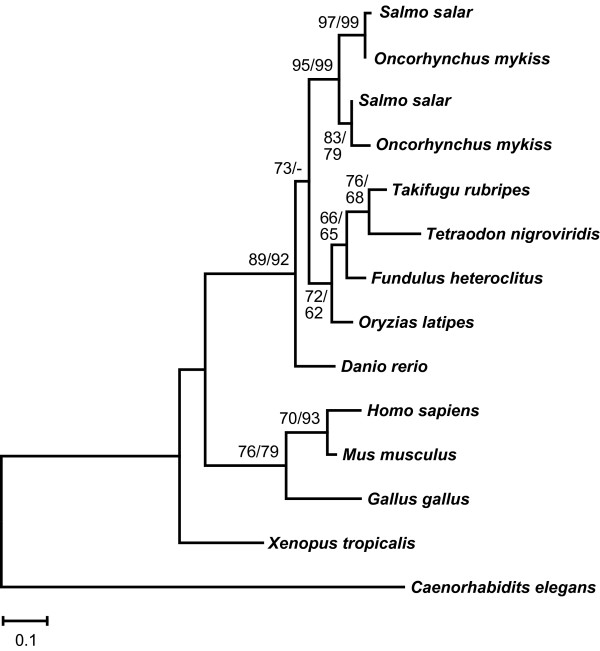
**Molecular phylogeny of the GchI feedback regulatory protein in vertebrates**. Maximum-likelihood phylogeny of Gchfr protein sequences based on 95 AA positions. The tree is rooted with Gchfr from nematode. Numbers at the branches denote bootstrap values (maximum likelihood/neighbor joining) above 50%. Gchfr is duplicated in salmon and rainbow trout due to the salmonid-specific tetraploidization.

The first group, termed *gchIa*, is phylogenetically well defined and includes the single *gchI *gene from mammals and birds and one copy from frog and teleosts including the known rainbow trout gene. All vertebrate *gchIa *orthologs are found within a region of conserved synteny. In rainbow trout, even two copies of *gchIa *are found, which might be the result of the salmonid autotetraploidization event that occurred 25 to 100 mya [30,31]. Paralogs of other pigment synthesis enzymes in trout and/or salmon (*silv, gchfr, dhprb, pam*; Figure 6,Table 1, [see Additional files 1, 2]) might also have resulted from this salmonid-specific genome duplication.

The second group of *gchI *genes, *gchIb*, consists of the second *gchI *gene from frog, the previously known zebrafish gene and further teleost genes. The orthology of *gchIb *genes is well supported by conserved syntenies between frog and teleosts. The third group, *gchIc*, has been found so far only in pufferfishes, stickleback and the gilthead seabream (*Sparus aurata*) and is also phylogenetically and syntenically well defined.

How are the three *gchI *groups related to each other? We confirmed by RHP mapping the chromosomal allocations of *gchIa *and *gchIb *in the zebrafish genome assembly on chromosomes 17 and 12, respectively. *GchIa *was assigned to LG 17 at a distance of 0.00 cR from marker fc19b04 with a LOD score of 18.4. *GchIb *was mapped to LG 12 at a distance of 0.00 cR from marker fc18g04 with a LOD score of 15.3. As LG 17 and LG 12 do not seem to have evolved by protochromosome duplication during the FSGD [33] and due to the presence of *gchIb *in amphibians, the duplication that led to *gchIa *and *gchIb *seems to be older than the split between ray-finned fishes and tetrapods. Both *gchIa *and *gchIb *genes are found in proximity to members of the *socs *gene family: *gchIa *is linked to *socs4 *in all vertebrates examined, *gchIb *to *socs5 *in frog and zebrafish (Figure 5a). *Socs4 *and *socs5 *are the closest related members within the *socs *gene family [46]. Therefore it seems most likely that *gchIa/b *and *socs4/5 *precursors were duplicated together, possibly during one of the two earlier rounds of genome duplication having taken place during the early evolution of the vertebrate lineage (1R or 2R) [30,47,48]. *Socs5 *is also found in mammals and birds within a syntenic region that resembles the *gchIb *region of frog and teleosts (Figure 5b) suggesting that *gchIb *was lost secondarily in these lineages. The human regions containing *GCHIA/SOCS4 *and *SOCS5 *are found on chromosomes 14 (Figure 5a) and 2 (Figure 5b), respectively, which were shown to contain many paralogous genes that arose during the 1R/2R genome duplications [48].

The origin of *gchIc *found in pufferfishes, stickleback and gilthead seabream remains unclear. Genes surrounding *gchIc *are not related to those of the other *gchI *regions and the human orthologs of these genes are found on chromosome 19q13. The corresponding chromosomal region on medaka chromosome 4 seems to be highly conserved in gene order with pufferfishes and stickleback, but a large gap is found in the medaka genome assembly between *pik3r2 *and *ankrd47 *(not shown). Thus, *gchIc *might also be present in medaka but absent from the current genome assembly. However, no EST or shotgun trace sequence from medaka was found that could represent *gchIc*. In zebrafish, a less conserved chromosomal block is found on chromosome 2. If *gchIc *genes arose as a paralog of *gchIa *or *gchIb *as result of the FSGD, one would expect to find *gchIc *on other chromosomes (Tetraodon: chr 14 or 3; medaka: chr 24 or 8; zebrafish: chr 20 or 3) [32-34]. Thus, there is no evidence that *gchIc *has been formed during the FSGD and its relationships to the other *gchI *groups remain elusive. It might be possible that *gchIc *arose by a lineage-specific gene duplication or that it is also a remnant of earlier rounds of genome duplication in vertebrates that has been maintained only in some teleost lineages.

The GchI enzymatic activity is regulated by the H_4_biopterin-dependent GTP cyclohydrolase I feedback regulatory protein (Gchfr) [49]. In most teleost species, a single *gchfr *gene was found. In contrast, two *gchfr *genes were identified in rainbow trout and Atlantic salmon (*Salmo salar*) (Figure 6). The phylogeny suggests duplication of *gchfr *in the common ancestor of these salmonid fishes, which fits well the salmonid autotetraploidization event [30,31].

#### 6-pyruvoyltetrahydropterin synthase and sepiapterin reductase

Subsequent steps of pteridine synthesis are catalyzed by the 6-pyruvoyltetrahydropterin synthase (Pts; EC 4.2.3.12) and the sepiapterin reductase (Spr; EC 1.1.1.153) (Figure 4). In the guppy, *pts *expression correlates with the presence of xanthophore-based yellow color patterns [50]. A single *pts *gene was found in all vertebrates analyzed including teleosts [see Additional file 5a].

**Figure 7:**
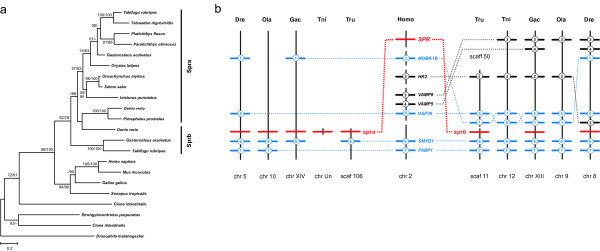
**Evolution of *sepiapterin reductase *genes in vertebrates**. (a) Maximum-likelihood phylogeny of Spr protein sequences based on 313 AA positions. The tree is rooted with Spr from fruitfly. Numbers at the branches denote bootstrap values (maximum likelihood/neighbor joining) above 50%. Groups are assigned according to synteny. (b) Synteny of *spr *regions in vertebrates. The human *SPR *region is syntenic to two *spr *paralogons in Takifugu (Tru), stickleback (Gac) and zebrafish (Dre). *sprb *was possibly lost in Tetraodon (Tni) and medaka (Ola). Numbered bars represent genes contributing to conserved synteny, genes that do not contribute to conserved synteny are not shown. Blue indicates genes that are duplicated along with *spr*. Dotted lines connect orthologous genes.

Sepiapterin reductase (Spr) catalyzes the next three steps towards H_4_biopterin (as well as a step in the third component pathway; see below). We could identify only one *spr *gene in tetrapods as well as in medaka and Tetraodon. In zebrafish, stickleback and Takifugu, however, two *spr *genes were found (Figure 7). Although the relationships between *spr *genes are phylogenetically not fully resolved, the genomic regions containing the duplicated teleost *spr *genes are syntenic to each other as well as to human chromosome 2, where *SPR *is found (Figure 7b). Both teleost *spr *genes are in close proximity to paralogs of *smyd1*. Additional duplicated genes are also found in these chromosomal regions. Furthermore, zebrafish chromosomes 5 and 8, which contain *spra *and *sprb*, respectively, evolved from the same pre-FSGD protochromosome [33,34]. It is therefore most likely that the *spr *duplicates found in some teleost lineages are remnants of the FSGD.

#### Enzymes of the H_4_biopterin regeneration pathway

**Figure 8:**
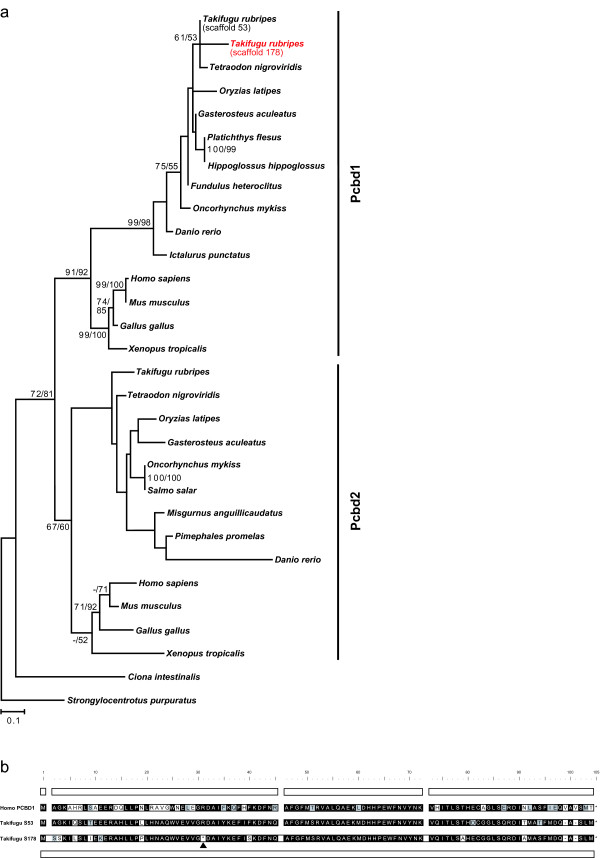
**Evolution of *pcbd *genes in vertebrates**. (a) Maximum-likelihood phylogeny of Pcbd proteins based on 107 AA positions. The tree is rooted with Pcbd from sea urchin. Numbers at the branches denote bootstrap values (maximum likelihood/neighbor joining) above 50%. Pcbd was duplicated in vertebrates (Pcbd1 and Pcbd2). In Takifugu, two *pcbd1 *are observed in scaffolds 53 and 178. The latter (red) is a retro-pseudogene. (b) Exon-intron structure of Pcbd1. Pcbd1 from human and Takifugu scaffold 53 consists of four exons indicated by 4 blocks. Takifugu scaffold 178 contains a "processed" pseudogene with a single exon (bottom row) and a premature stop codon (arrowhead).

The H_4_biopterin regeneration pathway involves the enzymes Pcbd (pterin-4 alpha-carbinolamine dehydratase/dimerization cofactor of hepatocyte nuclear factor 1 alpha (TCF1); EC 4.2.1.96) and Dhpr (Dihydropteridine reductase; EC 1.5.1.34) [25]. Pcbd is a bifunctional protein having a function as a dimerizing co-factor of the HNF1 homeobox transcription factors in addition to its enzymatic activity. A transcriptional target of the Pcbd/HNF1 complex is the *tyrosinase *promoter, pointing to a particular importance for melanophore differentiation in human [51]. In contrast to invertebrates, two *pcbd *genes, *pcbd1 *and *pcbd2*, were found in tetrapods and teleosts (Figure 8a), which is consistent with a previous analysis of this gene family [52]. Thus, *pcbd *was duplicated early in the vertebrate lineage, possibly during one of the two rounds of genome duplication (1R/2R). Two copies of *pcbd1 *were identified in Takifugu. While one copy (located in scaffold 53) has the usual structure with four coding exons, the other copy (scaffold 178) consists of a single exon (Figure 8b). A polyA sequence downstream of the stop codon and target site duplications indicative of a retrotransposition event were detected (not shown). Furthermore, a mutation in *pcbd1 *from scaffold 178 turned codon 31 into a premature stop codon (Figure 8b). Thus, this *pcbd1 *copy most likely represents a "processed" pseudogene generated by retrotransposition of a *pcbd1 *mRNA.

**Figure 9:**
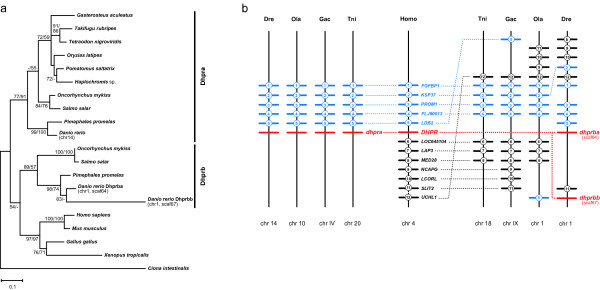
**Evolution of *dihydropteridine reductase *genes in vertebrates**. (a) Maximum-likelihood phylogeny of Dhpr protein sequences based on 247 AA positions. The tree is rooted with Dhpr from urochordates. Numbers at the branches denote bootstrap values (maximum likelihood/neighbor joining) above 50%. (b) Synteny of * dhpr *regions in vertebrates. The human *DHPR *region is syntenic to two paralogons in Tetradon (Tni), stickleback (Gac), medaka (Ola) and zebrafish (Dre). *Dhprb *was apparently lost in Tetraodon (Tni), stickleback (Gac) and medaka (Ola) and further duplicated in zebrafish, so that two duplicates, *dhprba *and *dhprbb*, are found on chromosome 1. Numbered bars represent genes contributing to conserved synteny, genes that do not contribute to conserved synteny are not shown. Blue bars indicate genes that are also duplicated. Dotted lines connect orthologous genes.

Several types of duplications have affected *dhpr *genes in teleosts (Figure 9). First of all, while a single *dhpr *gene is found in tetrapods as well as in pufferfishes, medaka and stickleback, two *dhpr *copies are present in salmon, trout and fathead minnow. The zebrafish genome contains even three copies of *dhpr*. Two major clades of *dhpr *genes become apparent in teleosts through phylogenetic analysis. The first clade, termed *dhpra*, consists of most of the teleost sequences including pufferfishes, medaka, stickleback, the zebrafish sequence found on chromosome 14 and one copy each of salmon, trout and fathead minnow. The second clade, *dhprb*, contains the second copy from salmon, trout and fathead minnow as well as the two zebrafish sequences found on chromosome 1 in scaffolds 64 and 67, respectively. These two zebrafish paralogs on chromosome 1 obviously arose by duplication in the family Cyprinidae and encode putative proteins that show 82% sequence similarity. Both copies of *dhprb*, termed *dhprba *(scaffold 64) and *dhprbb *(scaffold 67), could be amplified by PCR and sequenced from zebrafish cDNA with paralog-specific primer sets [GenBank: EF183528, EF183529], excluding genome assembly artifacts. In the zebrafish genome assembly, additional but partial sequences of *dhprba *and *dhprbb *are present in scaffold 63 and scaffold 67, respectively. These sequences were not included in further analyses.

The analysis of the *dhpr*-containing regions in vertebrate genomes revealed that the two main clades, *dhpra *and *dhprb*, might originate from the FSGD (Figure 9b): Genes surrounding the human *DHPR *gene on chromosome 4 are found in vicinity to the teleost *dhpra *gene (Tetraodon: chr 20; medaka: chr 10: zebrafish: chr 14) as well as on another chromosome (Tetraodon: chr 18; medaka: chr 1; zebrafish: chr 1). All these chromosomes evolved by duplication of the same protochromosome during the course of the FSGD [34]. Later on, *dhprb *was further duplicated in the lineage leading to zebrafish probably through intrachromosomal gene duplication. This led to the formation of *dhprba *and *dhprbb *on chromosome 1, where they are separated by approximately 1 Mb.

#### Enzymes involved in pteridine pigment synthesis

The third component pathway that leads to the formation of the yellow pteridine sepiapterin and its derivatives branches off from the first component pathway by hypothetical enzymatic reactions (Figure 4). Subsequent reactions require Spr (see above) and Xod/Xdh (xanthine oxidase/xanthine dehydrogenase; EC 1.17.3.2/EC 1.17.1.4) [25]. As in tetrapods, Xod/Xdh is represented by a single gene in teleost genomes [see Additional file 5b].

The biosynthetic pathway for the reddish drosopterin has not been elucidated yet in vertebrates and only one enzyme of the pathway in Drosophila, clot, has been characterized at the molecular level [53]. A single *thioredoxin-like 5 *gene, the vertebrate ortholog of Drosophila *clot*, is found in tetrapods and teleosts as well [see Additional file 5c].

Finally, the switch between the H_4_biopterin and sepiapterin synthesis might be regulated by PAM (protein associated with Myc), which is affected in the zebrafish *esrom *mutant that has reduced yellow pigmentation [54]. The *pam *gene is single-copy in teleosts and tetrapods [see Additional file 5d].

## Discussion

### Duplication of pigmentation genes: molecular mechanisms and evolutionary fates

In the present study, we have analyzed the two major pigment synthesis pathways in vertebrates, the melanin and the pteridine pathways, with respect to gene and genome duplications particularly within the teleost lineage. Seventeen vertebrate pigmentation genes were analyzed and various modes of duplication were observed. On the one hand, different rounds of genome duplication have expanded several pigment gene families. Five clear cases of FSGD-based duplications (*tyr*, *tyrp1*, *silv*, *spr*, *dhpr*) were found (29%). Other duplications might be the result of earlier rounds of genome duplication (1R/2R) [30,47] in the vertebrate stem lineage (*gchIa/b*, *pcbd1/2*). In addition, gene duplications generated by the recent salmonid-specific autotetraploidization [30,31] could be also detected (*silv*, *gchIa*, *gchfr*, *dhprb*, *pam*). On the other hand, lineage-specific local gene duplications were also identified: the duplication of *dhprb *in the zebrafish, the duplication by retrotransposition of *pcbd1 *in Takifugu and possibly the occurrence of *gchIc *in a common ancestor of pufferfishes, stickleback and perciforms. Although the majority of duplicated genes in vertebrate genomes were created by whole genome duplications [55], lineage-specific duplications of pigmentation genes, which have also been found for the urochordate *Ciona intestinalis *[29], seem to be a common theme in chordate evolution. In conclusion, teleost fishes have a greater potential repertoire of pigment synthesis genes than all other vertebrate groups. However, entirely duplicated synthesis pathways are not observed, and the function of both paralogs in pigmentation pathways remains generally to be demonstrated.

The impact of genome duplications on entire metabolic pathways in the vertebrate lineage has been studied so far only for the glycolysis [56]. Based on a similar approach to that used in the present study, the authors showed that none of the three rounds of genome duplication in the vertebrate lineage (1R/2R/FSGD) led to a completely duplicated glycolytic pathway in extant genomes. In total, 46% of the glycolytic enzymes in vertebrates were duplicated in teleosts due to the FSGD (11 of 24 enzymes) [56]. Here, 75% (3/4) of the melanogenic enzymes and 22% (2/9) of the enzymes from the pteridine pathway were found to be duplicated during the FSGD. Although the value for melanogenesis seems to be elevated in comparison to pteridine synthesis and glycolysis, all differences between the three pathways (glycolysis, melanogenesis and pteridine pathway) are statistically not significant (χ^2^-test, p > 0,05).

Generally, three different fates of duplicated genes are observed (reviewed by [10]). In most cases, one duplicate gets lost due to functional redundancy. This process of non-functionalization was estimated to have occurred in a range of 76% of FSGD duplicates in zebrafish [57] and 76 to 85% in the pufferfish lineage [33,39,58]. Here, for pigmentation genes 71% (12/17) were found to be reduced from two to one copy in teleosts after the FSGD but before the split of Ostariophysii (zebrafish) and Neoteleostei (medaka, stickleback and pufferfishes). Including lineage-specific losses, the ratio of non-functionalization for pigment synthesis genes is 82% (14/17) for pufferfish and medaka and 76% (13/17) for stickleback and zebrafish suggesting that pigment synthesis genes do not deviate from the global trend. Two other fates of gene duplicates might lead to the retention of both copies within a genome. Either one copy obtains a new function (neo-functionalization) or the original gene functions are divided between the two duplicates (sub-functionalization). Recently, it was shown that combinations of both mechanisms are possible (sub-neo-functionalization) [59]. Asymmetric evolution, which might be an indicator for neo-functionalization, has been observed for many duplicated genes in teleosts [58,60,61] including pigmentation genes [18]. Neo-functionalization of duplicated enzymes can lead, for example, to the evolution of new substrate specificities [62] or even of entirely new functions not associated with the enzymatic property [63]. Subfunctionalization of duplicated enzymes might occur at the level of gene expression leading, *e.g*., to tissue-specific expression ([56] and references therein) or at the protein level, when a duplicate becomes specialized for a certain substrate [64]. Whether and how functional divergence of duplicated pigment synthesis enzymes has occurred in teleosts will be an important focus of future studies.

### Evolution of the melanin synthesis pathway

The melanin synthesis pathway involves four enzymes. Three of them were found to be duplicated in teleosts as result of the FSGD. In the tyrosinase gene family, FSGD-duplication was observed for *tyr *and *tyrp1*, while *dct *was present as a single copy gene in all lineages analyzed. However, the retention of tyrosinase gene family members after the FSGD is variable between the different lineages (Table 1). *Tyra *was lost in the zebrafish and *tyrp1b *in the pufferfishes, while medaka and stickleback have retained both copies of *tyr *and *tyrp1*. Thus, the tyrosinase gene family is a good example for divergent resolution, *i.e*., differential loss of gene duplicates in divergent lineages, a mechanism that might facilitate speciation [13,65-67].

Mutational disruption of melanin synthesis at different steps of the pathway might lead to diverse forms of albinism [27]. Tyrosinase is the first, rate-limiting enzyme of melanogenesis. In the zebrafish, loss-of-function in the single *tyr *gene, *tyra*, leads to an albino phenotype [68]. In the medaka, several albino mutants were identified that are also affected in the *tyra *paralog [69]. Our data provide evidence for the presence of *tyrb *in the medaka but the functions of this paralog in teleosts remain unresolved. No *tyrb *mutant is available at present in fish. The fact that some *tyra *mutations in the medaka lead to a complete albino phenotype [69] suggests that *tyrb *cannot substitute for *tyra*. This is in agreement with functional studies of the two *tyr *duplicates in the rainbow trout [22]: simultaneous morpholino knock-down of both paralogs reduces pigmentation in the eye and the skin to the same amount as knocking-down *tyr *paralogs separately. Since knock-down of *tyrb *gene function in the rainbow trout leads to reduced pigmentation in the eye and the skin [22], *tyrb *seems to be involved in melanin synthesis too. Tyrosinase is involved in several steps of melanogenesis (Figure 1), and it is therefore possible that teleosts *tyr *paralogs might have become subfunctionalized and specialized for individual steps of the pathway.

There is so far no evidence supporting the functional divergence of *tyrp1 *paralogs in fish. Mutation of *tyrp1 *in mammals leads to reduced pigmentation [27]. No *tyrp1 *mutant has been identified in teleosts until today, possibly due to a functional redundancy of *tyrp1 *duplicates. Interestingly, in the present study a putative regulator of Tyrp1 function was also found to be duplicated in teleosts as result of the FSGD: *tyr *duplicates in teleosts are genetically linked to duplicates of *rab38 *(Figure 2b). Rab38 is thought to play a role in sorting Tyrp1 to the melanosome in mice [70].

The duplication of the *silver *gene has been previously described in the zebrafish [21]. Our study shows that this duplication is indeed the result of the FSGD and that *silver *has also been retained in duplicate in pufferfishes, medaka and stickleback. In zebrafish, *silva *is expressed in melanophores and the retinal pigment epithelium (RPE) of the eye, while *silvb *expression is restricted to the RPE [21]. The expression of *silv *paralogs is similar to the expression of duplicated *mitf *transcription factor genes [15]. In mammals, *Silv *transcription is dependent on Mitf [71,72]. It will be highly interesting to investigate the differential regulation of *silv *paralogs by Mitf duplicates in different teleost lineages.

Due to the limited knowledge of gene functions it remains elusive at present, whether there is a correlation between excess of genes involved in melanin synthesis and the vast diversity of coloration in fish. Functional experiments on the divergence of pigmentation gene duplicates are currently carried out in our laboratory to elucidate this question.

### Evolution of the pteridine synthesis pathway

The pteridine synthesis pathway has been less affected by the FSGD than the melanin pathway, but several cases of lineage-specific duplication were observed.

GchI is the first and rate-limiting enzyme of pteridine synthesis. In this analysis, we have observed an unforeseen diversity of *gchI *genes in vertebrates. We could identify two clades of *gchI *genes, *gchIa *and *gchIb*, which most likely arose through genome duplication during early vertebrate evolution, as well as a third clade of unresolved origin, *gchIc*, which is only found in some teleost species. The GchI enzyme is required at the initial step of the synthesis of both H_4_biopterin and pteridine pigments (Figure 4). GchIa has been found in all vertebrate lineages and is therefore most likely involved in H_4_bioterin formation. GchIb is only found in those lineages that possess xanthophores: teleost fishes and amphibians. Furthermore, *gchIb *from zebrafish, which is a paralog of the mammalian *gchIa *(and not its ortholog as previously thought), is expressed in the xanthophore lineage (but also in melanophores and neurons) [45]. We therefore propose that *gchIb *plays a major role in the synthesis of pteridine pigments of xanthophores and that it was lost secondarily in mammals and birds concomitantly to the loss of xanthophores in these lineages. Functional studies in teleosts and amphibians will be necessary to test this hypothesis.

Spr is involved in both the *de novo *synthesis of H_4_bioterin and the production of pteridine pigment after the split between both component pathways (Figure 4). Interestingly, the *spr *gene is found to be duplicated as result of the FSGD in zebrafish, stickleback and Tetraodon. It might be possible that each of the *spr *paralogs has become specialized for one component pathway, but expression data for duplicated teleost *spr *genes are not available at present. *Sprb *paralogs might have been lost quite recently in medaka as well as in Tetraodon after its split from Takifugu. This is a good example for the former observation that anciently duplicated genes still can be lost after millions of years [55].

Finally, *dhpr *in zebrafish illustrates how different evolutionary scenarios can progressively shape pigmentation gene families. After the duplication of *dhpr *in the FSGD, both *dhpr *paralogs were retained in Ostariophysii (zebrafish, fathead minnow) until *dhprb *was further duplicated in the zebrafish lineage, while *dhprb *was apparently lost from pufferfishes, medaka and stickleback.

### Evolution by genome duplication: the pigmentary system

The evolutionary significance of whole genome duplications is still widely debated. The two presumed rounds of genome duplication early in the vertebrate lineage (1R/2R) have been linked to an increase in phenotypic complexity and to the evolution of vertebrate-specific traits such as the neural crest [30,47]. Several authors have suggested that the divergent evolution of duplicates generated by the FSGD might be involved in species diversity in teleost fishes, which represent approximately 50% of all vertebrate species (reviewed in [10-13]). However, these hypotheses have been questioned based on the fossil record [73]. In addition, a reduced probability of extinction in teleost fishes compared to other vertebrates probably due to the FSGD has been proposed, since mutational robustness, increased genetic variation, and increased tolerance to environmental conditions could be by-products of genome duplication [74].

With regard to the pigmentary system, it has been previously suggested that the FSGD had a major importance for the evolution of pigmentation genes in teleost fish [18]. The present study puts further evidence in this direction by showing that pigment synthesis pathways (and the melanin synthesis pathway in particular) have been affected by the FSGD. Interestingly, the genetic repertoire for color perception, *i.e*., the *opsin *gene family, has also been expanded by duplications in the teleost lineage [75,76]. It remains to be elucidated whether the diversity and complexity of coloration observed in teleost fishes compared to other vertebrate groups are causally linked to the expansion of pigmentation gene families as result of the FSGD. This FSGD might also have provided the genetic raw material for the diversity of coloration within teleosts since species-specific sequence evolution of duplicated genes is a common mechanism in this group [61]. Furthermore, our study points out lineage-specific patterns of loss and retention of duplicated pigmentation genes in teleosts. Divergent resolution of duplicated genes might facilitate speciation events [13,65-67].

## Conclusion

The present study shows that teleost fishes have a greater repertoire of pigment synthesis genes than any other vertebrate group mainly due to the fish-specific genome duplication but also as result of other types of gene duplications. Thus, pigmentation genes from teleosts offer an excellent opportunity to study the effects of gene and genome duplication on gene regulatory, protein-protein interaction and metabolic networks (*e.g*., specification of chromatophore fates, receptor-ligand interactions and pigment synthesis, respectively) and their connections. Future studies on functional divergence of duplicated pigmentation genes will reveal important insights into the significance of gene and genome duplication for the evolution of vertebrate phenotypes.

## Methods

### Sequence database surveys

Nucleotide sequences of pigmentation genes from ray-finned fishes were identified using BLAST searches against GenBank (nr and EST databases), the current genome assemblies and Trace Archives at Ensembl [77] of zebrafish (Zv 6), medaka (version 1), Tetraodon (version 7), Fugu (version 4.0), stickleback (BROAD S1) as well as TIGR gene indices [78] of cichlids (*Astatotilapia burtoni*, *Haplochromis chilotes *and *Haplochromis sp*. 'red tail sheller'), catfish (*Ictalurus punctatus*), killifish (*Fundulus heteroclitus*), rainbow trout and salmon. Usually the human gene was used as query sequence. If necessary, coding sequences were annotated manually from genome assemblies based on sequence homology to other species. In some cases species-specific EST clusters were assembled. Similarly, sequences from human, mouse, chicken, frog, ascidian (*Ciona intestinalis*), sea urchin (*Strongylocentrotus purpuratus*), fruitfly (*Drosophila melanogaster*) and nematode *(Caenorhabditis elegans*) were obtained from GenBank or Ensembl under inclusion of information given in ref. 29.

### Sequence alignments and phylogenetic reconstructions

All nucleotide sequences obtained from BLAST searches were loaded into BioEdit [79], translated into proteins, and aligned using ClustalW [80] as implemented in BioEdit. Alignments were carefully checked and ambiguously aligned regions were removed prior to phylogeny analyses. Identical sequences were removed.

Larger draft neighbour-joining trees were obtained with MEGA3 [81]. Based on these trees, outgroups for final phylogenies were chosen. These were either the closest human paralog to the gene under investigation (in case of larger vertebrate gene families) or invertebrate orthologs.

Final protein maximum likelihood phylogenies were computed with PHYML [82,83] with 100 bootstrap replicates. Models of protein evolution and parameter values were determined with ProtTest [84]. PAUP [85] was used to obtain neighbor-joining bootstrap values of 10,000 replicates.

### Synteny analyses

Syntenic relationships between human and teleosts genomes within the chromosomal regions containing the gene of interest were inferred using the Reciprocal Blast Hit method [26].

Sequences of 15–20 genes surrounding the human ortholog were used as initial queries for BLAST searches against the five teleost genome assemblies at Ensembl [77], followed by reciprocal BLAST searches of the best hits against human and other teleost genomes.

### Radiation hybrid panel mapping

The zebrafish radiation hybrid panel LN54 [36] was used according to the supplier's instructions (Marc Ekker, University of Ottawa) to map *tyrp1a*, *gchIa *and *gchIb*. The following primer sets were used: Dre-tyrp1a-ex1F: 5'-ATGTTTGGACTTTATGGA GC-3', Dre-tyrp1a-ex1R: 5'-GTCAAACCCGCTGTAGTTC-3' (annealing temperature T_A_: 56°C); Dre-gchIA-ex1F: 5'-AAGAAACTGACGGAGCGATC-3', Dre-gchIA-ex1R: 5'-TCTCCTGGTATCCCTTGGTG-3' T_A_: 56°C); Dre-gchIB-ex1F: 5'-CAATGGCAAAATCGTCACAG-3', Dre-gchIB-ex1R: 5'-TGGTCTCGTGGTATC CCTTAG-3' (T_A_: 52°C). The obtained RHVECTORs were submitted to the LN54 radiation hybrid map website [86] to get chromosomal positions.

### Sequencing of Tetraodon *tyrp1b *pseudogene and zebrafish *dhprb *genes

The Tetraodon *tyrpb1 *pseudogene was amplified from genomic DNA and sequenced using primers Tni-ps-trp1b-F1 (5'- AACCTGGACACAAAGCCTCAC-3') and Tni-ps-trp1b-R1 (5'-ATGGTAGGAGAGAGCACGCAC-3') (T_A_: 62°C).

Zebrafish (strain WüAB) total RNA was extracted from various adult tissues using the TRIzol Reagent (Invitrogen, Karlsruhe, Germany). cDNA was synthesized with the RevertAid TM First Strand cDNA Synthesis Kit (Fermentas Life Science, St. Leon-Rot, Germany) and pooled. *Dhprb *sequences were amplified from the cDNA pool using paralog specific primer sets: Dre-dhprba-ex1F: 5'-CTCGTGAAGACAGAATGGCAG-3', Dre-dhprba-ex7R: 5' TGCTTTCTCCAGTCGTCCAC-3' (T_A_: 60°C); Dre-dhprbb-ex1F: 5'-AGCGAAGTAAAGAAAGTGATTG-3', Dre-dhprbb-ex7R: 5'-TAGGGGTAG CCACTGTTCTG-3' (T_A_: 58°C). PCR products were cloned using the TA Cloning Kit (Invitrogen, Karlsruhe, Germany) and subsequently sequenced. Sequencing was performed with a CEQ 2000XL system (Beckman Coulter, Krefeld, Germany).

## Authors' contributions

IB participated in the design of the study, carried out bioinformatic analyses and molecular studies and wrote the manuscript. MS was involved in the design of the study and helped to draft the manuscript. JNV participated in the design of the study, helped with data analyses and to draft the manuscript. All authors read and approved the final manuscript.

## Supplementary Material

Additional File 1 **Nucleotide accession numbers of melanin synthesis genes**. GenBank accession numbers, Ensembl accession numbers or TIGR EST clusters (TC) used for phylogenetic analyses are given. EST denotes manually assembled EST clusters. Partial sequences that were not included in final phylogenetic trees are indicated by ^#^, pseudogenes by ^ψ^. See Table 1 for species abbreviations. Click here for file

Additional File 2 **Nucleotide accession numbers of pteridine synthesis genes**. GenBank accession numbers, Ensembl accession numbers or TIGR EST clusters (TC) used for phylogenetic analyses are given. EST denotes manually assembled EST clusters. scaf: scaffold, ctg: contig of Ensembl genome assembly. Partial sequences that were not included in final phylogenetic trees are indicated by ^#^, pseudogenes by ^ψ^. See Table 1 for species abbreviations. Click here for file

Additional File 3 **Nucleotide phylogeny of *tyrp1 *genes in vertebrates**. Maximum-likelihood phylogeny of *tyrp1 *genes based on a 1681 nucleotide alignment (GTR+I+G model; parameter values estimated from the dataset). The tree is rooted with human *TYR *and *TYRP1 *genes. Numbers at the branches denote bootstrap values (maximum likelihood/neighbor joining) above 50%. The topology of the tree is consistent with the duplication of *tyrp1 *during the FSGD. Click here for file

Additional File 4 **Molecular phylogeny of melanosomal transporters: Oca2, Aim1, Slc24a5**. Maximum-likelihood phylogeny of (a) Oca2 (854 AA; rooted with Oca2 from sea urchin), (b) Aim1 (701 AA; rooted with human SLC45A1), and (c) Slc24a5 (651 AA; rooted with human SLC24A3/4 proteins). Numbers at the branches denote bootstrap values (maximum likelihood/neighbor joining) above 50%. No duplications were observed in teleosts. Click here for file

Additional File 5 **Molecular phylogeny of pteridine synthesis enzymes: Pts, Xod/Xdh, Tnxl5, Pam**. Maximum-likelihood phylogeny of (a) Pts (158 AA; rooted with Pts from *Ciona*), (b) Xod/Xdh (1453 AA; rooted with human AOX1; an Aox1 sequence from guppy (*Poecilia reticulata*) is wrongly annotated in GenBank as Xod/Xdh), (c) Tnxl5 (130 AA; rooted with Clot from *Drosophila*), and (d) Pam (4932 AA; rooted with Pam from *Drosophila*). Numbers at the branches denote bootstrap values (maximum likelihood/neighbor joining) above 50%. No duplications were observed in teleosts. Click here for file
