# Supplementary material for: Evolution of pigment synthesis pathways by gene and genome duplication in fish
Source: BMC Evol Biol. 2007 May 11;7:74. doi: 10.1186/1471-2148-7-74 (PMC1890551; doi:10.1186/1471-2148-7-74)
Supplement: Additional File 1 — Nucleotide accession numbers of melanin synthesis genes. GenBank accession numbers, Ensembl accession numbers or TIGR EST clusters (TC) used for phylogenetic analyses are given. EST denotes manually assembled EST clusters. Partial sequences that were not included in final phylogenetic trees are indicated by #, pseudogenes by ψ. See Table 1 for species abbreviations. [file 1471-2148-7-74-S1.pdf]

gene	zebrafish	medaka	Tetraodon	Takifugu	stickleback	other fishes	human	mouse	chicken	frog	outgroup
tyr a	NM_131013	AB010101	GSTENG0001<br>0318001	AF343911	ENSGACT00000<br>010570	Abr (AB178938)<br>Omy (AB122031)<br>Oni (AY333985)	NM_000372	NM_011661	NM_204160	CT025512	
tyr b	-	UTOLAPRE<br>05100115216	GSTENG0001<br>8906001	SINFRUT0000<br>0140542	ENSGACT00000<br>027278	Eha (AY333975)<br>Gpe (AY333979)<br>Ipu (AF216388)<br>Omy (AB117622)<br>Oni (AY333984)<br>Aba (AY333970)<br>Lpl (AY333982)					
tyrp1 a	XM_001344111	ENSORLT00<br>000005421	GSTENG0002<br>1333001	AF397401	ENSGACT00000<br>025846	Cau (S71755)<br>Omy (BX889446)<br>Oni (48249210)[#]<br>Ppr (EST)<br>Ssa (TC32296)[#]	NM_000550	NM_031202	NM_205045	NM_001016476	
tyrp1 b	BC076406	ENSORLT00<br>000005955	EF183530[#,ψ]	-	ENSGACT00000<br>021050	Man (EST)[#]<br>Ppr (EST)					
dct	NM_131555	ENSORLT00<br>000022235	GSTENT0002<br>6098001	AF397402	ENSGACT00000<br>005425	Cau (D63948)<br>Ipu (CV989943)<br>Omy (TC92526)[#]<br>Ssa (CO472055)	NM_001922	NM_010024	NM_204935	NM_001017161	
silv a	AY554264	UTOLAPRE<br>05100110606	GSTENG0003<br>3828001	SINFRUT0000<br>0138002	ENSGACT00000<br>000701	Hsp (BJ681186)[#]<br>Omy (CA358042)[#]<br>Ppr (EST)	NM_006928	NM_021882	NM_205112	BC075473	Hsa *GPNMB*<br>(NM_001005340)
silv b	AY864065	ENSORLT00<br>000020626	GSTENG0002<br>3380001	SINFRUT0000<br>0138004	ENSGACT00000<br>015380	Abu (DY631547)<br>Omy (CA379865)<br>Ssa (EST)<br>Ssa (BU694135)[#]					
oca2	XM_690715	AY605286	GSTENG0002<br>0137001	SINFRUT0000<br>0177655+SIN<br>FRUT0000016<br>2273	DW622716	Ame (DQ232591)	NM_000275	NM_021879	XM_425579	ENSXETT0000<br>0009006	Spu (XP_780555)
aim1	XM_001334037	AF332510	GSTENT0001<br>4448001	SINFRUT0000<br>0143778	ENSGACT00000<br>021547	Abr (AB178928)	AF172849	AF360357	XM_429218	NM_001011335	Hsa *SLC45A1*<br>(XP_089081)
slc24a5	AY538713	ENSORLT00<br>000003448	GSTENT0001<br>8071001+GST<br>ENT00018072<br>001	SINFRUT0000<br>0133125	ENSGACT00000<br>022163	Ppr (DT239784)	NM_205850	NM_175034	NM_001038497	ENSXETESTT<br>00000013901	Hsa *SLC24A3*<br>(NM_020689)<br>Hsa *SLC24A4*<br>(NM_153646)
