# Supplementary material for: Evolution of pigment synthesis pathways by gene and genome duplication in fish
Source: BMC Evol Biol. 2007 May 11;7:74. doi: 10.1186/1471-2148-7-74 (PMC1890551; doi:10.1186/1471-2148-7-74)
Supplement: Additional File 2 — Nucleotide accession numbers of pteridine synthesis genes. GenBank accession numbers, Ensembl accession numbers or TIGR EST clusters (TC) used for phylogenetic analyses are given. EST denotes manually assembled EST clusters. scaf: scaffold, ctg: contig of Ensembl genome assembly. Partial sequences that were not included in final phylogenetic trees are indicated by #, pseudogenes by ψ. See Table 1 for species abbreviations. [file 1471-2148-7-74-S2.pdf]

gene	zebrafish	medaka	Tetraodon	Takifugu	stickleback	other fishes	human	mouse	chicken	frog	outgroup
gchIa	XM_689781	scaf445: ctg95884	GSTENG00034788001	SINFRUG00000133569	DN694413	Hhi (DN794457)<br>Omy (Z49707)<br>Omy (CX026119)<br>Ppr (EST)	NM_000161	NM_008102	Z49267	NM_001006788	Cin (grail.884.2.1)
gchIb	AJ311846	BJ730802	GSTENG00005535001	SINFRUG00000154204	DT969946	Fhe (CN978857)[#]<br>Ppr (DT084998)	-	-	-	ENSXETESTT00000012596	
gchIc	-	-	CR687535	SINFRUG00000148390	scaf9: ctg3659	Sau (CB184157)	-	-	-	-	
gchfr	NM_200762	BJ909358	GSTENT00032398001	SINFRUT00000136686	ENSGACT00000008189[#]	Fhe (CN986362)<br>Omy (TC86934)<br>Omy (TC86933)<br>Ssa (TC31590)<br>Ssa (TC31222)	NM_005258	NM_177157	XM_420935	NM_213715	Cel (AC024761)
pts	AY692444	ENSORLT00000016473	GSTENT00013192001	SINFRUT00000178501	ENSGACT00000013540	Omy (TC72499)<br>Pfl (DV566143)<br>Ppr (EST)<br>Pre (AY034101)<br>Ssa (TC30535)	NM_000317	NM_011220	XM_417928	ENSXETT00000019399	Cin (grail.6.149.1)
spra	NM_001024430	ENSORLT00000014059	GSTENG00037979001	SINFRUT00000148435	DW638418	Ipu (CK419850)<br>Omy (TC87750)<br>Pfl (DV565949)<br>Pol (CX285140)<br>Ppr (DT236566)<br>Ssa(TC31725)	NM_003124	NM_011467	XM_423038	CX960701	Cin (grail.62.76.1)<br>Cin (genewise.116.150.1)<br>Spu (XM_784756)<br>Dme (NM_167157)
sprb	XM_001335582	-	-	U90880	ENSGACT00000008822	-					
xdh	XM_683891	ENSORLT00000001340	GSTENG00012569001	SINFRUT00000173565	ENSGACG00000015453	Fhe (EST)<br>Ppr (EST)<br>Pre (AY034103)<br>Sch (CO036809)<br>Ssa (EST)	NM_000379	NM_011723	NM_205127	ENSXETT00000047814	Hsa *AOX1* (NM_001159)

clot (txnl5)	BC078373	AM138077	CR715722	SINFRUT000 00162313	EG591107	Cca (CA968743)<br>Ipu (CK415949)<br>Omy (TC79005)<br>Ppr (DT181437)<br>Ssa (TC23297)	XM_083981	AJ344103	XM_415925	CT485728	Cin (S665)<br>Dre (NM_135086)
pcbd1	NM_200814	UTOLAPRE 05100108254	CR701296	scaf53: SINFRUT000 00131151<br>scaf178[ψ]: see Fig. 8b	ENSGACG0000 0003259	Fhe (TC5606)<br>Hhi (CF931918)<br>Ipu (DQ363469)<br>Omy (TC79784)<br>Pfl (DV570118)	NM_000281	NM_025273	NM_204905	ENSXETT00000 017961	Cin (S30)<br>Spu (XM_790053)
pcbd2	XM_001340481	ENSORLT00 000001056	GSTENT000 29908001	SINFRUT000 00142216	AC146538	Man (BJ820501)<br>Omy (TC93488)<br>Ppr (DT343367)<br>Ssa (TC27722)	NM_032151	NM_028281	NM_204203	ENSXETT00000 022848	
dhpra	XR_029166	ENSORLT00 000024958	GSTENT000 24438001	SINFRUT000 00153847	ENSGACT0000 0022011	Cca (CA966333)[#]<br>Hsp (TC1829)<br>Omy (TC76948)<br>Ppr (DT203527)<br>Psa (DY223262)<br>Ssa (DW531384)	NM_000320	NM_024236	NM_001006566	NM_001011492	Cin (AK114845)
dhprb	scaf64: XM_001331128<br>scaf67: XM_703196	-	-	-	-	Omy (TC71152)<br>Ppr (DT252845)<br>Ssa (EST)<br>Ssa (CK991195)[#]					
pam	NM_001012247	ENSORLT00 000014398	GSTENT000 27943001	SINFRUT000 00181449	ENSGACT0000 0002323	Abu (EST)<br>Hhi (EST)[#]<br>Omy (TC86133)[#]<br>Omy (CA344469)[#]<br>Ppr (EST)	NM_015057	AY325887	XM_417003	ENSXETG00000 015459	Dme (AF262977)<br>Spu (XM_784626)
