# Supplementary figures and images for: Evolution of pigment synthesis pathways by gene and genome duplication in fish
Source: BMC Evol Biol. 2007 May 11;7:74. doi: 10.1186/1471-2148-7-74 (PMC1890551; doi:10.1186/1471-2148-7-74)

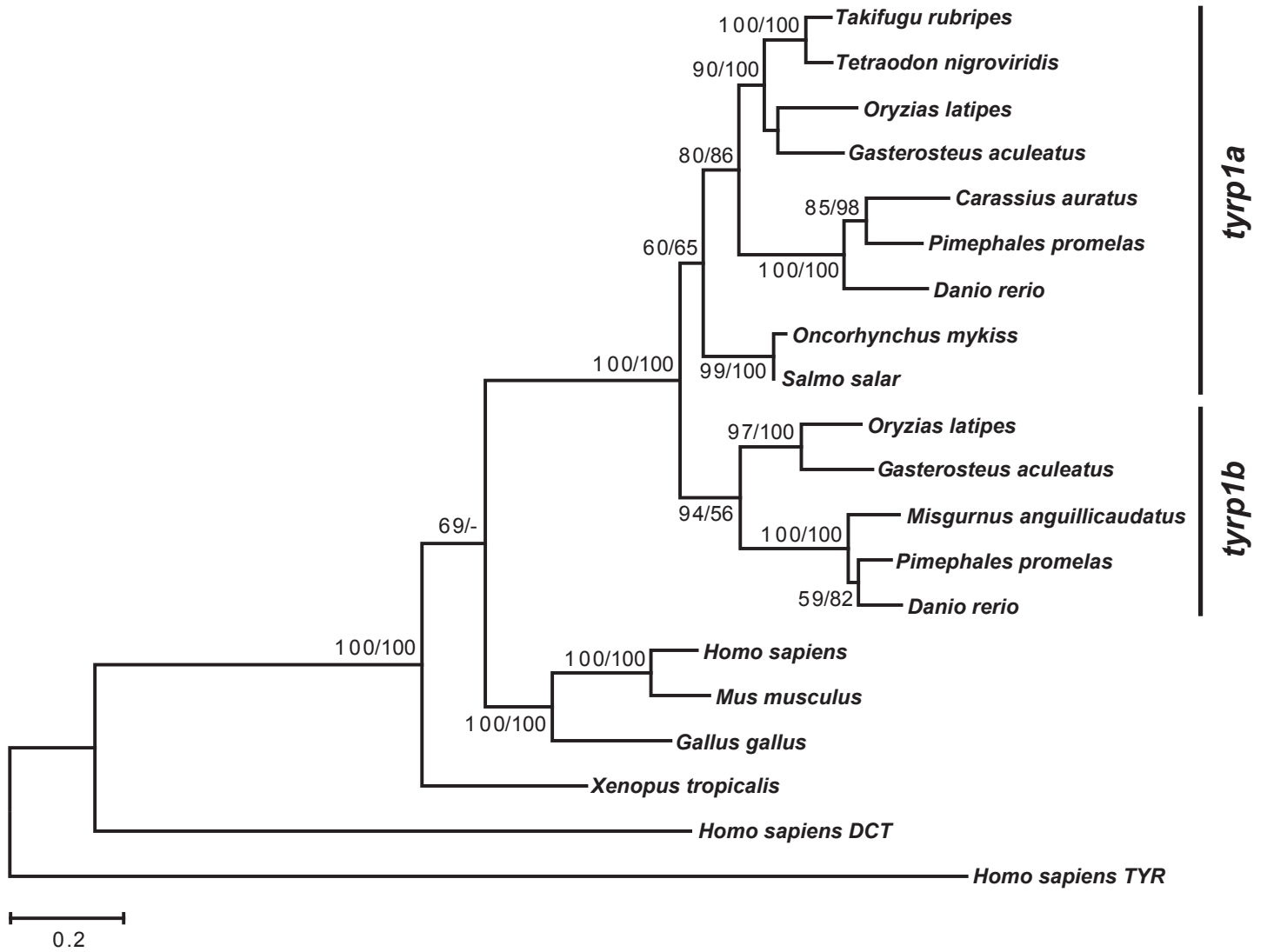

Supplement: Additional File 3 — Nucleotide phylogeny of tyrp1 genes in vertebrates. Maximum-likelihood phylogeny of tyrp1 genes based on a 1681 nucleotide alignment (GTR+I+G model; parameter values estimated from the dataset). The tree is rooted with human TYR and TYRP1 genes. Numbers at the branches denote bootstrap values (maximum likelihood/neighbor joining) above 50%. The topology of the tree is consistent with the duplication of tyrp1 during the FSGD. [file 1471-2148-7-74-S3.pdf]

a Oca2

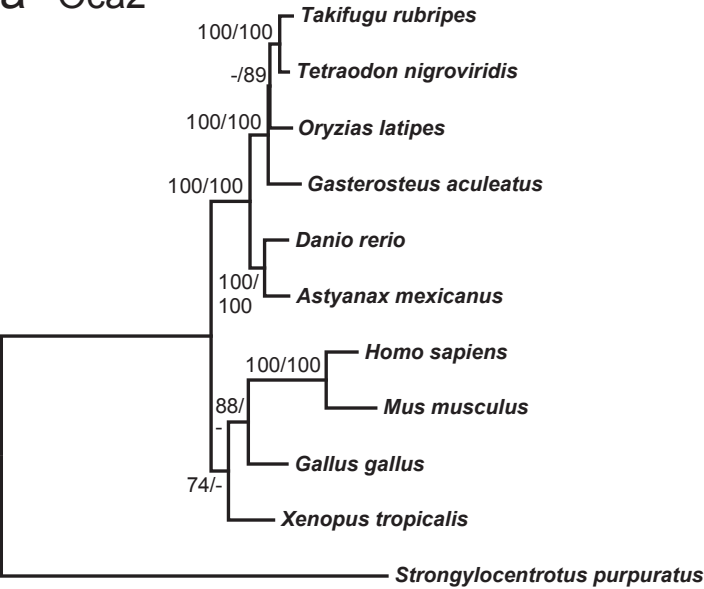

0.1

c Slc24a5

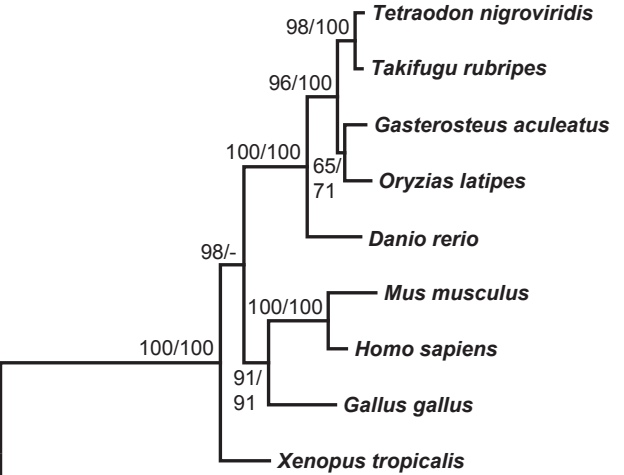

0.5

b Aim1

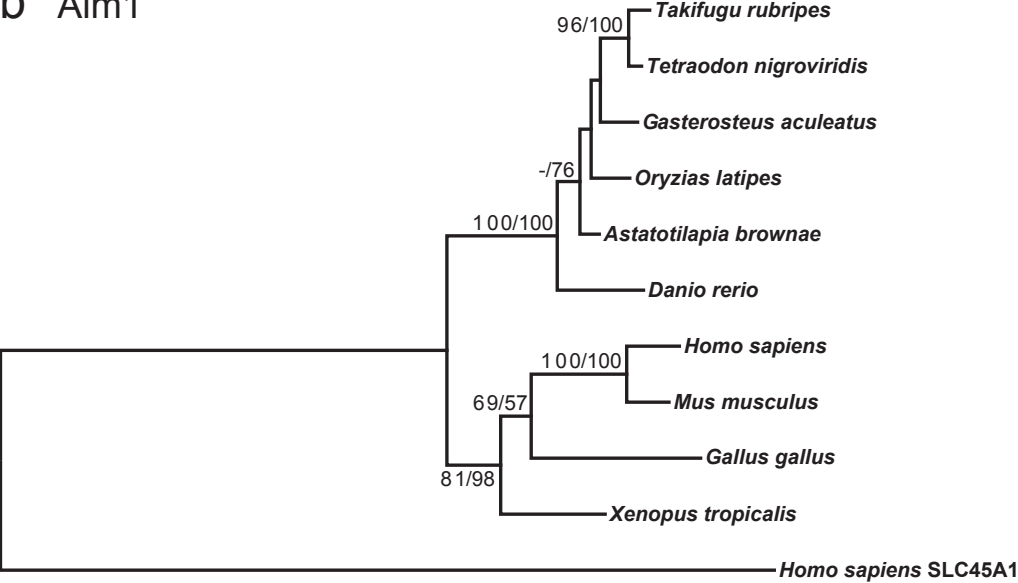

0.2

Supplement: Additional File 4 — Molecular phylogeny of melanosomal transporters: Oca2, Aim1, Slc24a5. Maximum-likelihood phylogeny of (a) Oca2 (854 AA; rooted with Oca2 from sea urchin), (b) Aim1 (701 AA; rooted with human SLC45A1), and (c) Slc24a5 (651 AA; rooted with human SLC24A3/4 proteins). Numbers at the branches denote bootstrap values (maximum likelihood/neighbor joining) above 50%. No duplications were observed in teleosts. [file 1471-2148-7-74-S4.pdf]

a Pts

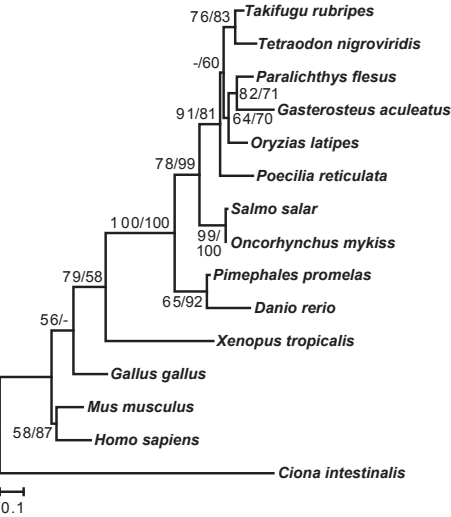

c Tnxl5

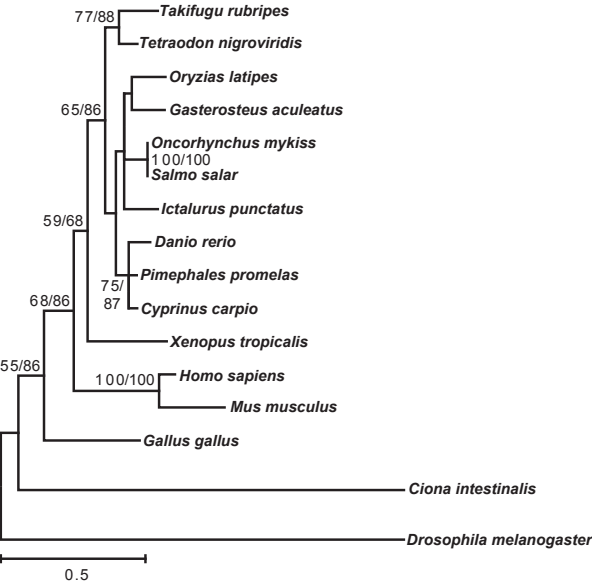

b Xod/Xdh

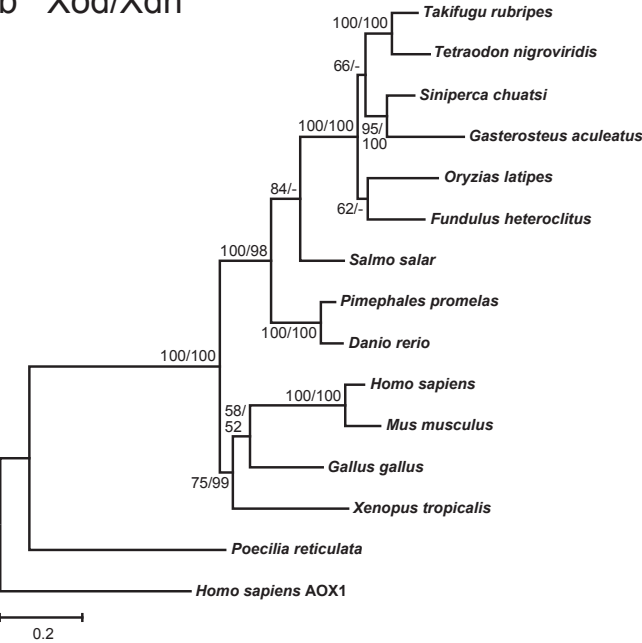

d Pam

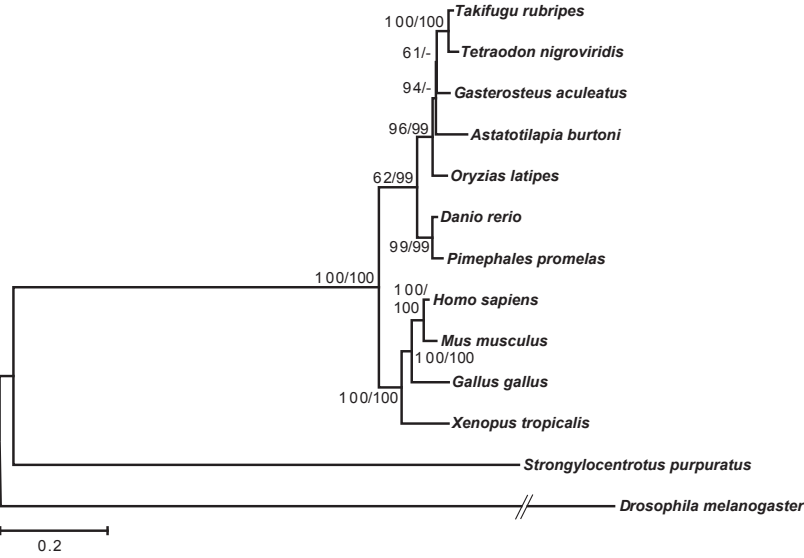

Supplement: Additional File 5 — Molecular phylogeny of pteridine synthesis enzymes: Pts, Xod/Xdh, Tnxl5, Pam. Maximum-likelihood phylogeny of (a) Pts (158 AA; rooted with Pts from Ciona), (b) Xod/Xdh (1453 AA; rooted with human AOX1; an Aox1 sequence from guppy (Poecilia reticulata) is wrongly annotated in GenBank as Xod/Xdh), (c) Tnxl5 (130 AA; rooted with Clot from Drosophila), and (d) Pam (4932 AA; rooted with Pam from Drosophila). Numbers at the branches denote bootstrap values (maximum likelihood/neighbor joining) above 50%. No duplications were observed in teleosts. [file 1471-2148-7-74-S5.pdf]
